# Farmer-Centred Multi-stakeholder Platforms: From Iterative Approach to Conceptual Embedding

**DOI:** 10.1007/s13132-023-01661-7

**Published:** 2024-02-08

**Authors:** Edith van Ewijk, Martha Ataa-Asantewaa, Kwabena O. Asubonteng, Yves P. B. Van Leynseele, Mercy Derkyi, Anna Laven, Mirjam A. F. Ros-Tonen

**Affiliations:** 1 Department of Geography, Planning and International Development Studies and Centre for Sustainable Development Studies, University of Amsterdam, Nieuwe Achtergracht 166, 1018 WV Amsterdam, The Netherlands; 2 Department of Natural Resources and Geo-Information Sciences, University for Development Studies, Nyankpala Campus, P.O. Box TL1882, Tamale, Northern Region Ghana; 3 School of Natural Resources, University of Energy and Natural Resources (UENR), P.O. Box 214, Sunyani, Ghana; 4 KIT Royal Tropical Institute, Mauritskade 63, 1092 AD Amsterdam, The Netherlands

**Keywords:** Farmer-centred MSPs, Knowledge co-creation, Inclusive learning, Relational learning, Innovations ‘from below’, Ghana

## Abstract

Research on knowledge exchange in multi-stakeholder platforms (MSPs) within smallholder farmer settings has primarily focused on adaptive designs and increased innovation rates. This paper addresses a knowledge gap by providing insights into the limited understanding of how knowledge sharing in MSPs can be inclusive of smallholder farmers’ knowledge. It shares experiences with a farmer-centred approach to learning and knowledge-sharing with smallholder farmers in Ghana and places this iteratively developed approach within a theoretical perspective. We relate the farmer-centred approach to five design principles inspired by human-centred design rules and inclusive development dimensions. Findings from observations, interviews, and focus group discussions illustrate how the farmer-centred approach creates multifunctional spaces for peer-to-peer learning, interaction with institutional actors, and network building (design principle 1). Prior farmer-centred research ensured that themes addressed were relevant to participant farmers and brought their innovations ‘from below’ to the table (design principles 2 and 3). A majority representation of farmers, facilitation by local researchers, and organising platform meetings at the district level created a safe space for farmers to share their knowledge and feel included and empowered (design principle 4). Several local institutional actors took ownership of the platform principles, which created opportunities for their manifestation in future collaborations with farmers (design principle 5). However, challenges remain in engaging crucial actors from higher scale levels and reducing donor dependency. The design principles presented in this paper contribute to the genuine inclusion of farmers’ knowledge and innovations. We recommend government agencies at the national and district levels incorporate these design principles into their work plans and budgets and prioritise farmers’ knowledge and innovation capacity for inclusive learning and knowledge co-creation.

**Supplementary Information:**

The online version contains supplementary material available at 10.1007/s13132-023-01661-7.

## Introduction

To address persistent challenges related to poverty, food insecurity, unsustainable farming, and climate change in agricultural landscapes, donors and scholars are increasingly collaborating to co-create knowledge through multi-stakeholder platforms (MSPs) (Bitzer & Marazzi, 2021; Braun et al., 2021; Tengberg et al., 2021). The MSP concept is based on the understanding that ‘wicked problems’ (Rittel & Webber, 1973) require a combination of codified scientific and context-embedded experiential knowledge (van Ewijk & Baud, 2009)^1^ and learning across disciplines and sectors (Dentoni et al., 2018; Herens et al., 2022; Reed et al., 2019). MSPs, particularly innovation platforms, are also initiated to encourage innovation and the adoption of agricultural practices (Adolwa et al., 2017; Klerkx et al., 2012; Schut et al., 2018). These platforms bring farmers together with public and private actors to exchange knowledge and ideas for joint learning, action, and problem-solving (Cullen et al., 2014; Ros-Tonen et al., 2015). MSPs recognise that farmers’ local knowledge, experiences, and own initiatives fundamentally shape agricultural innovations and can trigger peer-to-peer learning between farmers (Adolwa et al., 2017; Dabire et al., 2017; Weyori et al., 2018). This paper focuses on the inclusion of smallholder farmers’ local knowledge in knowledge exchange and joint learning in MSPs. We adopt Shaw and Kristjanson’s definition of ‘joint learning’ as ‘a change in understanding that goes beyond the individual to become situated within wider social units or communities of practice through social interactions between actors within social networks’ (Shaw & Kristjanson, 2014, p. 2686). Knowledge co-creation occurs when different kinds of knowledge are combined, resulting in new knowledge (Akpo et al., 2015; Struik et al., 2014). Through knowledge exchange, joint learning, and knowledge co-creation, MSPs aim to change behaviour, practices, policies, and institutions to enhance innovation and farmers’ livelihoods (Cullen et al., 2014; Van Ewijk & Ros-Tonen, 2021). Such a combination of different knowledges and knowledge-sharing methods can help address both ‘tangible’ issues (e.g. soil erosion) and non-tangible issues (e.g. marketing) (Musvoto et al., 2015).

A recent systematic literature review conducted on MSPs in sub-Saharan Africa (Van Ewijk & Ros-Tonen, 2021) revealed that most MSPs are donor-driven and embedded in (action) research (Braun et al., 2021). Preceding MSP meetings, stakeholder analysis and diagnostic studies are often carried out to identify relevant actors and concerns (e.g., Adjei-Nsiah & Klerkx, 2016; Adjei-Nsiah et al., 2008; Adu-Acheampong et al., 2017; Röling, 2010; Van Paassen et al., 2013) to ensure that platform activities are demand-driven and address the needs of participants (Kilelu et al., 2014; Nyikahadzoi et al., 2012; Schut et al., 2016). However, these studies did not or hardly pay attention to the inclusivity of knowledge exchange from the perspective of smallholder farmers. Despite the growing recognition of the importance of including local knowledge (e.g. Akpo et al., 2015; Shaw & Kristjanson, 2014), it is still insufficiently clear how MSPs can be genuinely inclusive of smallholder farmers’ knowledge. This study addresses this knowledge gap by presenting, analysing, and conceptualising our experiences with a farmer-centred MSP approach field-tested in Ghana as part of a transdisciplinary, collaborative research project (see the methodology section). We believe the lessons are also relevant for other areas in sub-Saharan Africa where MSPs are being implemented. The farmer-centred approach discussed in this study is an inclusive and relational approach towards knowledge exchange and co-creation with a crucial role for smallholder farmers in their engagement with peers, institutional actors,^2^ and researchers. In this paper, we link this iteratively developed approach to principles of design thinking research (Plattner et al., 2015) and inclusive development theory (Gupta et al., 2015; Hickey et al., 2015) to give the approach a more robust conceptual basis.

Design thinking builds on participatory action research (Faure et al., 2014; MacDonald, 2012) to find innovative and creative solutions to problems from a human-centred perspective (von Thienen et al., 2018). It emphasises ‘the human perspective, human dignity and human rights in all steps of the problem-solving process’ (Val et al., 2017: 760). Inclusive development theory prioritises marginalised people and sectors and considers social, relational, and environmental inclusiveness to promote human well-being, sustainability, and empowerment (Gupta et al., 2015). By combining these concepts, our farmer-centred approach views MSPs as potentially inclusive when they (i) prioritise farmers’ livelihood orientations, knowledge, experiences, capabilities, and innovation capacity in knowledge exchange processes; (ii) create a space for farmers’ empowerment and self-determination; and (iii) consider sustainability and landscape concerns beyond the farm level (c.f. Ros-Tonen et al., 2019). This study explores how our experience of creating a safe and inclusive space for smallholder farmers to voice their concerns, share their knowledge and innovation capacity, and learn jointly from interactions aligns with design thinking and inclusive development principles. After outlining the methods used in this paper, we define five design principles based on human-centred design rules and inclusive development dimensions. These design principles provide a framework to analyse the farmer-centred MSPs in the results section. Next, we discuss what worked and why and the limitations and challenges of the approach. The concluding section synthesises the findings and makes suggestions for further research.

## Methodology

### Project Background and Study Sites

The farmer-centred MSP approach documented in this paper was developed by a transdisciplinary consortium of academic and non-academic partners, including NGOs and government agencies, as part of the Inclusive Value Chain (VCC) project funded by NWO-WOTRO Science for Global Development in the Netherlands.^3^ The project, which included three PhD and two post-doc projects, was conducted in Ghana and South Africa from 2014 to 2020 and involved organising MSPs targeting smallholder farmers. The data analysed in this study was collected as part of the Putting Heads Together project, funded by the Science for Using Research (SURe) programme, another call within NWO-WOTRO Science for Global Development.^4^ The project aimed to examine knowledge exchange and joint learning in three agricultural and food-related multi-stakeholder partnerships, including the Inclusive VCC project (discussed in this paper), the Treefarms project^5^ (covered in the results section under principle 5), and the Food and Business Knowledge Platform in the Netherlands, which played a role in formulating the research calls that funded both projects.

**Fig. 1:**
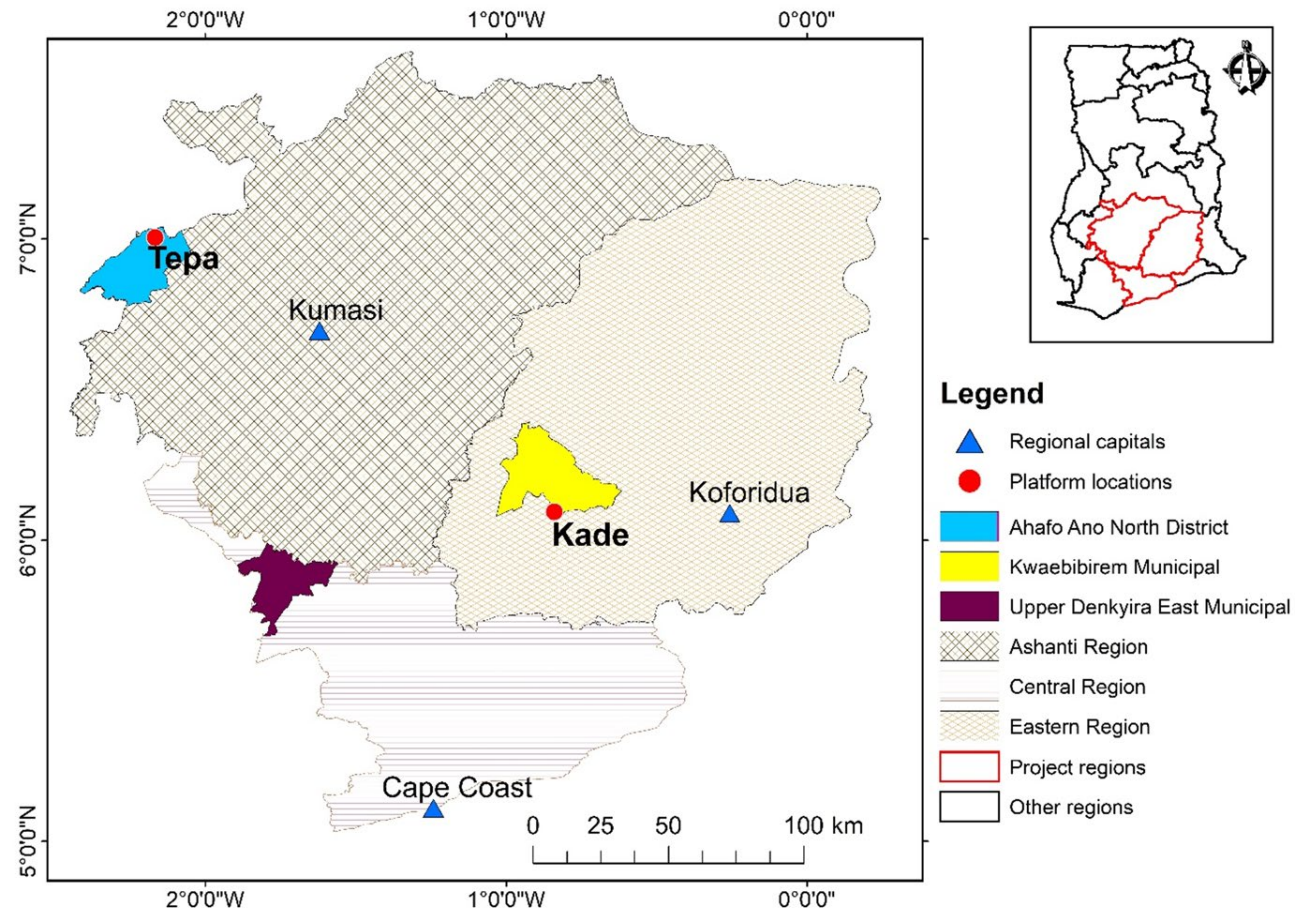
Study sites of the Inclusive Value Chain project in Ghana. (*Source:* compiled based on information from https://data.humdata.org/dataset/ghana-administrative-boundaries for the boundaries of administrative regions)

The Inclusive VCC project was initiated by practitioners in the consortium who observed that value chain collaborations with smallholder farmers often failed to match their realities and ambitions (Ros-Tonen et al., 2015, 2019). Therefore, the project aimed to unravel the diversity of smallholders and corresponding differences in their constraints, opportunities, and aspirations to make value chain collaborations more inclusive of female and other marginalised farmers.^6^ The research focused on the cocoa and oil palm sectors in Ghana,^7^ where smallholder farming is prevalent (Laven, 2010; Osei-Amponsah et al., 2012; Asubonteng et al., 2018), and value chain collaborations have been developed to enhance smallholders’ access to inputs and high-end markets (Ros-Tonen et al., 2015). The project was conducted in the Ashanti Region (the cocoa-dominated Ahafo-Ano North District), the Eastern Region (Kwaebibirem Municipal, where both cocoa and oil palm are grown), and the Central Region (the oil-palm dominated Upper Denkyira East Municipal) (Fig. 1). This selection allowed us to include both cocoa and oil palm farmers.^8^

The consortium of Dutch and Ghanaian academic and practitioner organisations organised MSPs through annual learning platforms, bringing together researchers, practitioners, and farmers for knowledge exchange, joint learning, and co-creation. The platforms aimed to provide a safe space for farmers to express their concerns, interact with actors within and outside the value chain, and learn from low-cost and accessible innovations ‘from below’ (Jaskiewicz & Laven, 2016) developed by peers to overcome daily challenges. Unlike innovation platforms described in the literature (e.g. Audouin et al., 2021; Klerkx et al., 2013; Nederlof & Pyburn, 2012; Schut et al., 2016, 2018), the primary objective was not to increase productivity or encourage the adoption of externally developed innovations but rather to foreground the knowledge and innovation capacity of farmers. The learning platforms were held in Tepa (Ahafo-Ano North District, Ashanti Region) and Kade (Kwaebibirem Municipal, Eastern Region) (see Fig. 1).

### Data Collection and Sampling Methods

This study utilised a qualitative research design, including the data collection methods specified in Table 1. First, researchers participated in and observed MSP meetings, where they acted as facilitators and presented preliminary research findings. Second, they observed and participated in project team meetings. Third, in-depth interviews and focus group discussions were conducted. Fourth, validation and dissemination workshops were held. Fifth, project documents were analysed. These methods were also utilised to collect data on the Treefarms project, which is discussed in the section on cross-over learning under design principle 5.

**Table 1:** Overview of research activities, methods and participants and their links to the projects in Ghana

**Research activities Putting Heads Together project**	**Related to the MSPs organised in the Inclusive VCC project**	**Related to the MSPs organised in the Treefarms project**	**Total**
Observation and participation in MSPs	- 2 learning platforms (Tepa and Kade, July 2018) - 1 dissemination workshop (Accra, October 2019)	- 1 community of practice meeting (Kumasi, November 2018) - 1 learning platform (Nyinahin, April 2019) - 1 dissemination workshop (Kumasi, April 2019)	6 meetings
Observation and participation in project team meetings	4 meetings	3 meetings	7 meetings
In-depth interviews	15	8	23
Focus group discussions	1 (8 participants)	1 (3 participants)	2 (11 participants)
Validation and dissemination workshops	2 (35 participants)	1 (10 participants)	3 workshops
Document analysis	28 project documents	16 project documents	44 project documents

Source: Compiled by the authors

The in-depth interviews were conducted with purposively selected respondents, namely the project partners and lead actors involved in the projects (*n* = 23) in 2018–2019. The interviews aimed to gather information on several topics, including (i) the interviewees’ background characteristics and their role in the project; (ii) their previous experience with capacity building and knowledge sharing; (iii) their experiences, roles, and opinions regarding capacity building and knowledge sharing; (iv) what they learned from the project; (v) whether and how they shared these lessons within and outside their organisation; and (vi) their perceptions of the potential and challenges of MSPs. The interview guide is available in the supplementary material.^9^

Data on farmers’ perspectives of MSPs was collected at the end of each MSP meeting in which they participated. The Inclusive VCC project held seven MSP meetings in Ghana, one in Accra in 2015, and three yearly meetings in Tepa and Kade each from 2016 to 2018. Farmer participants were selected from a database of 168 tree-crop farmers developed through a baseline survey at the start of the project. The database contained details such as farmers’ names, contact information, and the tree crops they cultivated. The second author of this paper played a key role in the selection process, ensuring that the selection was fair and that both male and female farmers, different age categories, and different farmer profiles^10^ were represented. In addition, chief farmers,^11^ who represent farmers in their communities in dealing with buyers and institutional actors, were invited because of their key roles in the communities, including knowledge dissemination. Farmers who had participated in the research prior to the meeting were also invited. Although the proportion of farmers participating in the MSPs was relatively large (more than 50%, up to 85%) and represented a diverse group in terms of gender, age, farmer profile, and communities, only a limited number of farmers in the area could join due to the limited budget and the size of the venues. The average size of platform participants was about 50.

In addition, a focus group discussion was conducted with eight farmers who were identified as ‘changemakers’ during the research preceding the learning platform meetings and who had taken a leading position in their communities after engaging in a learning platform (see the section on design principle 1 in the results). Finally, two workshops were held 1 year after the last MSP meetings to validate and disseminate the research findings. A total of 35 participants, purposefully selected to have a representative sample of MSP participants, attended these workshops, of which 20 were farmers.

### Data Analysis

The observations during MSPs, interviews, focus group discussions, and meeting reports were transcribed and manually coded using the design principles outlined in the previous section as codes. All co-authors were actively involved in the organisation, evaluation, and facilitation of the platform meetings and preceding research. They discussed and validated findings during staff meetings, informal occasions, and two ‘write shops’. The lead author performed the initial analysis to reduce the risk of researcher bias.^12^ She was more distant from the project under investigation because she joined it only in its final year and was not involved in setting up the Inclusive VCC project. To reduce participant bias, we triangulated the results with data from validation workshops and post-project interviews by external project evaluators. The evaluation documents, made available to the authors but not publicly available, confirmed the findings in this paper.

## Theoretical Background: Design Principles for Farmer-Centred MSPs

We derived the design principles for farmer-centred MSPs from two bodies of literature: human-centred design thinking and inclusive development theory. Human-centred design thinking has primarily been applied in engineering, industrial design, teaching, and information and communication technology (Kolko, 2018; Plattner et al., 2015). Four human-centred design rules can be readily applied to farmer-centred approaches. The *human rule* emphasises empathy and a human-centred perspective. It asserts that people are at the core of all innovations and that innovations should aim to solve problems to meet human needs. This rule also recognises the social nature of innovations. The *ambiguity rule* establishes that innovations are founded on experimentation and iteration. It encourages actors to remain open to multiple solutions and to challenge their assumptions. The *tangible rule* states that the ‘innovator’s story’ should be made tangible, context-specific, and user-specific. It should be communicated concretely so that stakeholders can easily understand and relate to it. The *redesign rule* stresses the importance of learning from past experiences and outcomes. It encourages actors to examine how problems have been addressed previously, by whom, and to what effect (Meinel & Leifer, 2015).

Aside from aiming to adopt a human-centred approach to knowledge sharing and learning, we also strived for an inclusive approach. Therefore, we developed the second set of design principles for farmer-centred MSP meetings based on the social, relational, and environmental dimensions of inclusive development theory (Gupta & Vegelin, 2016; Gupta et al., 2015; Ros-Tonen et al., 2019). The *social dimension* emphasises concern for the well-being of poor and marginalised people, inclusive learning, innovation, and co-creation based on the recognition of local knowledge and innovation capacity, and alignment with smallholders’ realities, including their opportunities, constraints, and aspirations. The *relational dimension* focuses on empowerment by addressing the concentration of power and inequality. In the context of knowledge exchange, it involves efforts to overcome expert-led ‘epistemic oppression’ and overvaluation of ‘outside, authoritative knowledge’ over place-based, experiential knowledge of resource-poor farmers (Leta et al., 2018, p. 474). Finally, the *environmental dimension* promotes sustainability and resilience (Gupta & Vegelin, 2016; Ros-Tonen et al., 2019). It acknowledges smallholders’ interest in pursuing cost-effective, sustainable farming methods, enhancing their control over production factors, and sustaining endogenous knowledge systems (Van der Ploeg, 2008).

**Fig. 2:**
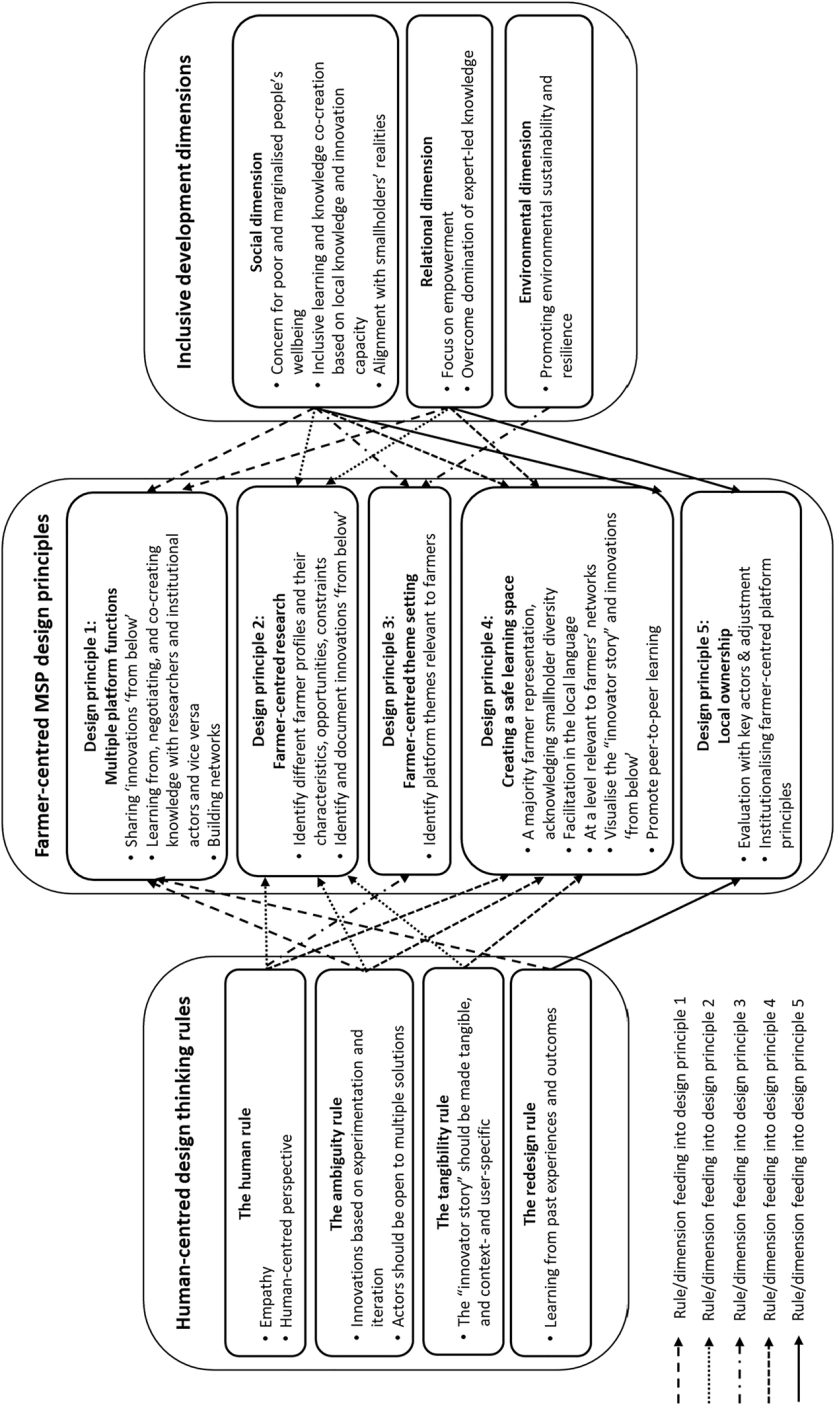
Farmer-centred MSP design principles based on human-centred design rules and inclusive development dimensions

We integrated the human-centred design rules and inclusivity dimensions into five principles for a farmer-centred approach, as shown in Fig. 2. These principles emerged and were refined through an intuitive and iterative process of learning by doing, in which feedback from farmers and other stakeholders played a crucial role, consistent with the redesign rule. For instance, at the end of each platform meeting, we solicited participants’ feedback, and the project team and institutional partners held a meeting the following day to evaluate the event.

The first and overarching design principle acknowledges the *multiple roles* of MSPs as spaces where farmers share their innovations, engage with researchers and institutional actors, and learn from researchers, who can use the meetings to validate research results. These meetings also enable all actors to build networks.

The second principle focuses on *farmer-centred research*, which provides insight into farmers’ diverse realities, constraints, and opportunities, particularly focusing on low-cost and low-tech innovations ‘from below’. These innovations are designed, experimented with, and implemented by farmers to tackle daily livelihood and environmental challenges (Jaskiewicz & Laven, 2016; Ros-Tonen et al., 2019; Waters-Bayer et al., 2015).

The third design principle of *farmer-centred theme setting* refers to identifying themes relevant to farmers’ daily livelihood and environmental challenges. The fourth principle is to create a *safe and empowering learning space* where male and female farmers can freely express themselves and interact with their peers, institutional actors, and researchers. Key to creating such a safe space is ensuring a majority representation of farmers, facilitating in the local language, organising platform meetings at a level relevant to farmers, and visualising ‘innovator stories’ (Meinel & Leifer, 2015, p. 4) and innovations ‘from below’. It is also important to acknowledge the heterogeneity among smallholder farmers and ensure that this diversity is reflected in MSP participants to ensure that diverse needs and livelihoods are considered.

Finally, the *local ownership* principle emphasises the importance of documentation, evaluation, and redesign to learn from past experiences with local stakeholders. This approach promotes the creation of local ownership of the farmer-centred platform principles, which is necessary if local stakeholders wish to carry the platforms further. Generally, this requires involvement from actors at higher scale levels, such as national government bodies.

These five principles differ from other MSP initiatives reported in the literature that link knowledge co-creation to productivity, income increase, and uptake of technologies developed by ‘experts’ (Holzmann et al., 2014; Pyburn & Woodhill, 2014). Although these initiatives acknowledge the importance of including local knowledge, our approach places local knowledge at the centre stage and foregrounds the quality of knowledge negotiation processes as a goal in itself. Our farmer-centred approach goes beyond studying the effectiveness of adoption-focused agricultural extension (c.f. Stewart et al., 2015) and aims to redesign platforms to include smallholder farmers’ knowledge, promote social learning, and establish local learning systems (c.f. Leta et al., 2018).

## Results^13^

In this section, we analyse how MSPs can become more inclusive of smallholder farmers’ knowledge by applying the farmer-centred design principles shown in Fig. 2. Our analysis is based on the iterative approach developed and field-tested in Ghana.

### Design Principle 1: Multiple Platform Functions

The MSPs in the Inclusive VCC project fulfilled multiple functions. They facilitated peer-to-peer and cross-sector learning; facilitated farmers’ engagement with government agencies, value chain actors, and researchers; helped researchers to validate and disseminate research findings; and forged networks.

A key objective of the MSPs in the Inclusive VCC project was to facilitate *peer-to-peer learning*, and we observed several instances of farmers adopting knowledge from their peers. The low-cost and low-tech innovations ‘from below’ were generally easy to apply. Examples of such innovations include the introduction of organic manure and mulching, the use of plant-based extracts as pesticides, the formation of saving groups, the pruning of cocoa trees, and the diversification of farming practices.

Peer-to-peer learning was enabled by identifying female and male ‘changemakers’ and conducting on-the-spot interviews with them. Changemakers are individuals who bring new ideas that impact other people’s lives and often ‘do things differently’ (Laven et al., 2017, p. 3; see also Jaskiewicz & Laven, 2016). They may include farmers and processors who demonstrate alternative ways of organising production or finding ways to access savings, credit, or markets. They may also include buyers, extension officers, and researchers who found new and unique ways of interacting with farmers (Laven et al., 2017, p. 3).

Live or video-recorded interviews with changemakers were shown to farmers, highlighting how their peers dealt with day-to-day challenges. Many farmers reported having taken up lessons learned from their peers, resulting in increased yields and income. For example, at the validation and dissemination workshop in December 2020, a farmer recalled learning about the importance of diversification at a learning platform meeting in 2016. However, he was initially hesitant to take risks. In 2017, he learned about soap making and selling from a female farmer at a learning platform meeting and decided to take it up. With the income generated from the soap-making business, he was able to buy a motorised tricycle (*aboboya*) which he used to transport harvested food crops.

Another objective of the MSPs was to incorporate farmers’ and other actors’ knowledge to foster *cross-sector learning and engagement*, particularly between farmers and institutional actors, to contribute to farmer-centred policymaking. During MSP meetings, government agencies had ample opportunity to explain policies and clarify their roles to farmers, while farmers could ask questions, voice their concerns, and hold government agencies accountable. This also contributed to better coordination among the various agencies, which rarely met each other and sometimes conveyed conflicting messages in the past. For instance, in the Tepa area, farmers received conflicting messages from the Ministry of Food and Agriculture and the Forestry Commission about the number of trees they could plant on their cocoa farms, leading to confusion. However, during a learning platform meeting, the Forestry Commission clarified that farmers could plant up to 18 trees per hectare of land.^14^

Another widely adopted lesson introduced by institutional actors and researchers was tree planting. A policy officer from the Forestry Commission mentioned that immediately after a learning platform meeting, where information was given on tree planting and the tree registration process,^15^ many farmers came for seedlings and requested the registration of trees they had planted on their farms.

The researchers aimed to prioritise farmers’ knowledge in both the research design and MSP meetings. They encouraged the farmers to actively participate and contribute their knowledge, facilitating the *dissemination and validation of research findings* and stimulating knowledge exchange.

A farmer from Kade likened the learning platforms to a knowledge market, where knowledge was exchanged fairly between farmers, researchers, and institutional actors. Another farmer, who also served as a purchasing clerk for Armarjaro Ghana Limited, a licensed cocoa-buying company, further elaborated on this comparison:

These practitioners tend to bully us with their knowledge because they are more educated than us farmers, and coming to their office was even a challenge for us. However, with the learning platforms, we were at the same level (farmer and purchasing clerk AGROECOM Ghana Ltd [Armajaro], validation workshop Tepa, December 2020).

The local knowledge of farmers was highly valued by institutional actors and researchers, who even adopted some of the farmers’ innovations. In interviews with government agencies and NGOs, it was mentioned that they incorporated this local knowledge from farmers into their extension work or changed their approach to training and workshops. This involved providing more opportunities for discussions and deliberations with farmers instead of the traditional one-way knowledge transfer.^16^

Despite adopting some farmers’ knowledge or learning platform principles, government agencies and NGOs did not fundamentally change their working plans or incorporate institutional and organisational changes. At the district level, government bodies are required to implement policies from national governmental bodies and are often not allowed to make changes on their own. However, the national government bodies were not consistently engaged in the platform meetings, which likely contributed to the rigidity of the working plans and institutional ‘stickiness’ (Pierson, 2000).

As discussed in the section on design principle 4, organising the MSPs at the district level—where farmers’ professional networks evolve and institutional actors operate but rarely meet one another—was essential for facilitating *network-building* among farmers and enhancing the engagement of actors working closely with them. While various institutional actors were aware of the presence of certain organisations, they felt a lack of personal contact. The farmer-centred MSPs helped establish contacts across organisations that work with farmers, enhancing their engagement with them. For example, a staff member of the Oil Palm Research Institute (OPRI) in Kade referred to the creation of a ‘family of institutions’, stating that several of them had started to contact each other directly and include each other in programmes targeting farmers in the municipality (validation workshop held in Kade, December 2020). An officer of the Ministry of Food and Agriculture (MoFA) said that after attending the learning platform meetings, he began directing farmers with cocoa-related questions to his contact at the Cocoa Health and Extension Division of the Ghana Cocoa Board (CHED-COCOBOD). Previously, he would have responded that cocoa was not within MoFA’s domain (interview with the MoFA officer, October 2019).

Representatives of government agencies and public institutions considered the knowledge brought in by the researchers as relevant, particularly for raising awareness on sustainability issues and landscape changes. An extension officer explained that one of the researchers’ presentations on landscape changes was so memorable that no one would soon forget the images and the explanations (Asubonteng et al., 2018). While identifying several farmer profiles and gender roles might not seem surprising at first glance, these insights were eye-opening for institutional actors who often focused on translating general agricultural policies and organising extension meetings for large groups of farmers.

### Design Principles 2 and 3: Farmer-Centred Research and Theme Setting

Conducting research prior to each platform meeting was an essential feature of our farmer-centred approach. A well-trained research team led by the KIT Royal Tropical Institute carried out fieldwork for 5 days per community, with financial support from the Lindt Cocoa Foundation, to identify key themes of concern to farmers and their self-designed innovations^17^ to meet these challenges (see Supplementary Material 2). Following human-centred design principles (see Fig. 2), the methodology facilitated an inclusive data-collection process. The fieldwork involved transect walks, focus group discussions with women and men, and mixed focus group discussions. The Ghanaian PhD researchers in the project generated and disseminated insights into farmers’ heterogeneity, livelihood trajectories, and struggles with food insecurity, tenure, and landscape dynamics (Asubontenget al., 2018, 2020, 2021; Ataa-Asantewaa, 2023). These insights into farmers’ realities facilitated alignment with their opportunities and constraints.

Identifying and documenting innovations ‘from below’ was critical in preparing for the learning platform meetings. These innovations were brought to the meetings and disseminated through live interviews and video documentation, enabling the incorporation of farmers’ experiential knowledge in a tangible way, in line with the tangibility rule in human design thinking. The research also helped identify themes relevant to farmers (design principle 3), allowing them to contribute their knowledge related to these themes and learn from their peers.

### Design Principle 4: Creating a Safe Learning Space

A critical decision in designing the MSPs was to prioritise the creation of a safe and empowering learning space for farmers. This was achieved by ensuring that most participants were farmers, with a relatively equal representation of both males and females. This approach strengthened their position and empowered them by providing a platform to share their knowledge, experiences, and concerns.^18^

Having a farmer majority was crucial to boosting farmer confidence and overcoming subordination in hierarchical relationships with government officers. We observed this during the learning platform meetings, and both farmers and institutional actors confirmed this in informal interactions at the end of the MSPs. Female and male farmers felt safe to speak out, raise their voices, and ask questions openly to institutional actors, providing evidence of space for critical perspectives. We also observed that other farmers, female and male, spoke openly. With such a majority, farmers felt free to voice their concerns and, in some cases, to disagree with institutional actors, knowing they would receive support from peers who faced similar challenges. For example, during the learning platform in July 2018, a fierce discussion arose about an oil palm company’s corporate social responsibility activities in Kade. A farmer strongly disagreed with a private sector actor about the company’s social responsibility projects in his community. Almost all the farmers supported their colleague, corroborating the issues raised. Farmers criticised the company for taking away farmland and not keeping their promises, such as providing or maintaining services like access to clean water, which were part of its corporate social responsibility policy.

The Ghanaian researchers involved in the project were critical in creating safe spaces. They spoke the same language as the farmers and institutional actors (Twi) and were familiar with the farmers, having built trust through their longstanding presence in the field. This position enabled them to act as knowledge brokers between farmers and other actors during the learning platform meetings and be accepted as trustworthy facilitators.

Organising learning platform meetings at the district level was crucial for promoting peer-to-peer learning and knowledge uptake. One farmer in Kade highlighted the stark contrast between the ‘boring’ first meeting held in Accra, which focused primarily on expert knowledge, and the subsequent learning platforms organised at the district level. According to the farmer, the later learning platforms were especially useful as they provided practical knowledge that participants could apply immediately upon returning home (validation workshop in Kade, Ghana, December 2020).

Creating a safe learning environment and foregrounding farmers’ knowledge and innovative capacity aimed to empower them and enhance their networks. Our research uncovered various instances where farmers expressed feeling empowered. One farmer stated:

The Ministry of Food and Agriculture and Armajaro [AGROECOM Ghana Ltd] kept telling us that farming is like a business, but I never saw it that way. However, coming to the learning platform every year and meeting other stakeholders who value our knowledge as farmers made me feel like an expert in my trade. Since then, I started planning and keeping records of my farming, and now I calculate things like a businessperson (farmer participant in the Tepa validation workshop, Ghana, December 2020).

Another farmer explained:

For me, it was the fact that we women were included, and we could voice out during the programme. So many of us women have tried many things after the learning platforms, and it is all thanks to the fact that we felt included and recognised (farmer participant in the Kade validation workshop, Ghana, December 2020).

These quotes exemplify how participating in the learning platforms and being recognised for their knowledge and skills can change farmers’ perspectives on farming, enabling them to feel more confident in their abilities.

Inspired by the learning platform, one female farmer formed a women’s ‘susu group’ (a saving group), which helped them get timely access to farm reinvestment loans and create other livelihood opportunities. As a result, this farmer was recognised as a changemaker and shared her experience with other farmers, who were inspired to organise themselves and establish similar groups.

Interviews with farmers and institutional actors and observations revealed that the learning platform meetings empowered farmers by giving them a platform to showcase their knowledge and innovations. Additionally, the meetings provided a space for effective engagement and discussion with institutional actors, contributing to farmers’ sense of empowerment.

### Design Principle 5: Local Ownership

The MSPs organised as part of the Inclusive VCC project, like most agriculture and food-related MSPs in sub-Saharan Africa, were established by researchers in close collaboration with practitioners and supported by donor funding. Sustaining the learning platforms was not an aim of the programme from its onset.^19^ However, after each learning platform meeting in the Inclusive VCC project, an evaluation session was held on the following day with key actors representing farmers and other participants to discuss what went well and what could be done differently. Additionally, researchers and practitioners documented all meetings and made reports available to project partners who expressed an interest in adopting the learning platform concept.^20^ Researchers also compiled a concept note on the fundamental principles and methodology for organising learning platforms (Laven et al., 2017) and designed a learning platform brochure.^21^ The latter was richly illustrated with photos and disseminated among farmers, who greatly appreciated it. Furthermore, researchers supported the first steering committee meetings in Tepa and Kade, consisting of institutional actors and farmers who wanted to institutionalise or sustain the learning platform locally.^22^ Although several project partners took ownership of some of the platform principles (see the following sub-section) and created more space for exchange with farmers and aligning policies, sustaining the MSPs after project closure was challenging.

Project partners felt that the consortium had not put enough effort into developing a gradual exit strategy to support local steering committees in sustaining the learning platform concept. The partners were critical of the donors and believed the consortium could have done more to assist the local committees in securing funds to organise platform meetings after the project’s closure. As a policy officer expressed it:

We will still need small support. It could be by way of capacity building; how did they come by those resources? Teach us, so we can also fetch and get it too (Interview with CHED policy officer, October 2019).

In 2019, the University of Energy and Natural Resources (UENR) organised fundraising and proposal writing workshops for the executive committee members established in Sunyani and Tepa to continue the learning platforms. The Association of Commonwealth Universities (ACU-UK) funded the workshops under its CIRCLE uptake programme. During a validation meeting in Tepa (December 2020), recommendations on sustaining the platforms were shared, including (i) government agencies adopting the approach and incorporating it into their annual work plans and budgets, (ii) making the learning platforms community-based with chief farmers playing an active role, (iii) using social media platforms such as WhatsApp, as many farmers have mobile phones, and (iv) increasing the use of peer-to-peer (farmer-to-farmer) learning.

An unexpected outcome of the Inclusive VCC project was the cross-over learning that took place with the Treefarms project, which was also funded by NWO-WOTRO and partly operational in the same region during the same period. Some team members from the Inclusive VCC project were also involved in the Treefarms project, which facilitated knowledge-sharing between the two initiatives. More details on this cross-over learning are discussed in the following sub-section.

#### Cross-Over Learning

The Treefarms project, which was coordinated by the Resource Management Support Centre (RMSC) of the Ghana Forestry Commission, aimed to generate knowledge and build capacity for the successful integration, production, and marketing of shade-tolerant non-timber forest products (such as black pepper, grains of paradise, and honey) in forest reserves and off-reserve tree farms. The project was active in several forest districts in the Ashanti Region (including the Nkawie and Mankranso Forest Districts) and the Brong Ahafo Region (now Ahafo Region) (including the Goaso Forest District).

The RMSC adopted and institutionalised the learning platform concept after its coordinator participated in the Inclusive VCC project’s learning platform meetings. She lobbied to adopt the learning platforms in her organisation’s work plans, including the research carried out prior to the meetings. UENR researchers who participated in both projects facilitated cross-over learning and taught the learning platform method to RMSC staff. KIT from the Netherlands, which was also involved in the Inclusive VCC project, provided support.^23^

The implementation of the farmer-centred approach by RMSC revealed some interesting similarities and differences in applying design principles. Regarding *design principle 1* (multiple platform functions), we observed that knowledge co-creation was stronger in the Treefarms project than in the Inclusive VCC project. We attribute this to the fact that researchers in the Treefarms project were staff members of the organisations involved who pursued their MPhil degrees at UENR as part of the project’s capacity-building component. This ensured that the knowledge obtained through the research could be immediately implemented. For example, research on the wood used for beehives was carried out by a staff member of the Forest Services Division of the Forestry Commission in Kumasi, supervised by a lecturer at UENR. The study built on farmers’ observations that some beehives remained uncolonised, and this was related to the wood species used for the hives; bees stayed away from beehives made of *Cedrela odorata* (Okyere-Amoateng & Abugre, 2021). An NGO representative engaged in the Treefarms project subsequently expressed:

We always thought beehives are just a thing. But then in the community people told us that the type of wood determines how many bees are attracted into the beehives… it was community knowledge (Interview with NGO representative, June 2018, Accra).

Another example is the assumption that black pepper is a shade-tolerant species, which farmers’ experiential knowledge debunked. While black pepper can survive under shade, it needs some sunlight to grow. As a result, the Treefarms project had a stronger knowledge negotiation and co-creation function. However, sharing ‘innovations from below’ and networking were less prominent due to the project’s lesser focus on research before platform meetings (*design principle 2*). The RMSC had adapted the format for collecting innovations ‘from below’ to a more condensed version, as they did not have the capacity to spend 5 days per community to conduct and document the research.

The theme of the platform meetings in the Treefarms project was determined by the Forestry Commission’s objective to promote the introduction of shade-tolerant species in reforestation schemes, indicating the absence of farmer-centred theme setting (*design principle 3*). Although most participants were farmers, the meetings were facilitated in Twi, and the innovations ‘from below’ were visualised through videos, creating a safe learning space (*design principle 4*) to empower farmers to speak out and learn from their peers’ knowledge and innovations was less emphasised compared to the Inclusive VCC project. The primary focus was to obtain buy-in for integrating non-timber forest products in reforestation schemes.

Local ownership (*design principle 5*) was reflected in the inclusion of the farmer-centred MSP approach in the RMSC’s 2-year work plan and budget. However, no evaluation with key actors or adjustments could be observed as the concept was only implemented once at the end of the Treefarm project.

The example of cross-over learning notwithstanding, the adoption of the farmer-centred MSP approach was limited in terms of ownership and sustainability. Although some project partners adopted farmer-centred platform principles selectively, there were no clear indications at the time of writing this article that these principles would be widely adopted in the study areas.

## Discussion

In this section, we will discuss the added value and limitations of the farmer-centred MSP approach regarding the five design principles and the broader literature, aiming to draw lessons for future MSPs and research. Additionally, we will critically reflect on the role of engaged scholars in this process.

### The Added Value and Limitations of a Farmer-Centred MSP Approach

This paper demonstrates that MSPs can serve multiple functions (*design principle 1*). By promoting exchange between farmers and government agencies, NGOs, and companies, MSPs can help harmonise and clarify institutional policies (Johansson et al., 2013). In this way, MSPs act as bridging spaces (Berkes, 2009; Hahn et al., 2006). This is particularly important for smallholder farmers, who often receive conflicting messages from institutional actors (Mwase et al., 2015; Wortmann et al., 2020). Several studies have reported on this function of MSPs, often observing that organisations had little interaction before the platforms were established (Röling, 2010; Nederlof & Pyburn, 2012; Van Paassen et al., 2014).

Joint learning and knowledge co-creation are the goals of most MSPs. However, many platforms still have a one-way transfer of knowledge (Cullen et al., 2014; Pyburn & Woodhill, 2014; Schut et al., 2016, 2018). In the farmer-centred approach discussed in this paper, farmers’ knowledge and innovations take centre stage in their interaction with peers and other actors (*design principles 1, 2, and 4*). Our study demonstrated that the adoption of knowledge and practices such as tree planting and using organic manure aligned well with farmers’ environmental concerns. This supports findings from other research that MSPs can potentially address environmental and landscape issues (e.g. Dabire et al., 2017; Johansson et al., 2013; Totin et al., 2018).

The research team was surprised by the evidence of peer-to-peer learning (*design principle 4*), given that the meetings were organised only once a year. The literature mentions continuous engagement and participation by farmers as preconditions for knowledge and innovation uptake (Betge et al., 2020; Dabire et al., 2017; Weyori et al., 2018). While increased yields or income resulting from MSPs are also reported in the literature (e.g. Téno & Cadilhon, 2017; Vissoh et al., 2017), they often stem from a series of MSPs with a well-defined focus on one topic (e.g. a specific crop) (Schut et al., 2016). Two factors may explain the uptake despite the lack of frequent interactions. The first is the project’s application of the innovations ‘from below’ concept, which spoke to farmers’ imagination and was easily adopted due to its relevance and proximity to their daily lives, budgetary constraints, and technological capacities, as reported by the farmers and institutional actors who participated in this study. The second is the majority presence of farmers, as demonstrated by Pamuk and Van Rijn (2019: 1242), who found that innovation platforms with a diverse range of stakeholders are less effective in promoting the adoption of agricultural innovations.

The research conducted prior to MSP meetings, aimed at identifying changemakers and innovations ‘from below’, placed farmers at the forefront of the process (*design principle 2*). This approach differs from diagnostic studies in other MSPs, which lay the groundwork for stakeholder analyses or identify production and institutional constraints to the uptake of innovations (Adjei-Nsiah & Klerkx, 2016; Adu-Acheampong et al., 2017; Van Paassen et al., 2013). Our research was farmer-centred, emphasising their realities, ambitions, constraints, and creative ways of coping with them. By bringing their innovations ‘from below’ to the table, other farmers learned how to tackle daily livelihood and environmental challenges through low-cost and low-tech solutions. Similar farmer-centred or farmer-led research preceding platform meetings has been reported for the Promoting Local Innovation (Prolinnova) network (Waters-Bayer et al., 2015) and the Netherlands Land Academy (LANDAC) (Betsema et al., 2018). However, the Prolinnova network mainly focused on technological innovations (e.g. water and soil conservation or land reclamation) and rarely highlighted social and institutional innovations (Waters-Bayer et al., 2015), unlike our research findings (see also Jaskiewicz & Laven, 2016). In LANDAC, the research served to identify fundamental issues or challenges rather than farmer-led innovations.

The concept of innovations ‘from below’ has not gained much traction in the literature. However, one exception is Van Damme et al. (2014), who perceived such innovations in Rwanda as either local adaptations of top-down interventions or resistance against those. Based on our study, we believe that innovations ‘from below’ emerge from farmers’ constraints and possibilities rather than a deliberate act of resistance, as framed in agroecology literature (Gliessman et al., 2019; Holt-Giménez & Altieri, 2016).

Prior research identified relevant topics from a farmer’s perspective and their knowledge of them, ensuring that the platform themes aligned with their realities and concerns (*design principle 3*). A demand-driven approach that caters to the participants’ needs is also considered essential in broader MSP literature (e.g. Kilelu et al., 2014; Nyikahadzoi et al., 2012; Schut et al., 2016).

*Design principle 4* aimed to create a safe and empowering learning space by ensuring a farmer majority representation, facilitation in the local language, and organising the platform meetings at the district level. This approach enabled all participants to speak out and voice their concerns. Other studies have also reported the importance of using the local language (Akpo et al., 2015). However, the literature has hardly addressed the importance of farmer representation and their dominant share in the total number of MSP participants. This study demonstrated that having at least half of the MSP participants being farmers was essential for them to gain confidence in raising their voices.

The study’s results on ownership and adoption of farmer-centred platform principles (*design principle 5*) demonstrated promising cross-over effects but limited internalisation and structural policy change. The findings regarding increased cross-sectoral collaboration might be temporary, as Ghana’s institutional environment does not encourage collaboration across agencies. Such collaboration is often stimulated by external donors and actors (Dabire et al., 2017; Ragasa et al., 2016; Schut et al., 2018). These findings indicate the limited reach and scope of science-driven research designs and align with studies of other agriculture and food-related MSPs in sub-Saharan Africa (Van Ewijk & Ros-Tonen, 2021).

Despite the added value of the farmer-centred approach, there were also limitations and challenges. One downside of being closely connected to farmers’ lifeworld is the limited interactions with actors at higher scale levels, such as ministries at the national level. However, such interactions are necessary to address persistent challenges related to sustainable farming, food insecurity, environmental degradation, and climate change. Fostering cross-scale interactions is often beyond the scope of researcher-steered MSPs, as seen in a systematic literature review of agriculture- and food-related MSPs in sub-Saharan Africa (Van Ewijk & Ros-Tonen, 2021). This project was no exception to this challenge.

Another limitation of the project was the limited number of MSP meetings organised, which were held only once a year and in a few locations. During the Inclusive VCC project’s dissemination workshop in Accra (October 2019), participants suggested organising MSPs more frequently and at the community level to reach more actors. However, most research projects lack the funds and staff capacity to do so, and this project was no exception. Although researchers continued meeting farmers while still in the field, interactions after ending fieldwork and follow-up in-between meetings were limited due to financial constraints. These limitations relate to the critical constraint of donor dependency in research-steered MSPs, as discussed previously. Such limitations directly affect the inclusivity dimensions that inform the farmer-centred MSP design principles as they limit the MSP’s scope and the farmers and locations that can be reached.

### Limitations of this Study

This study has some limitations that should be acknowledged. First, there may be a participant bias in the data gathered through interviews with institutional actors and farmers, as respondents might provide answers that they think would please the researcher or are socially acceptable. Additionally, such interviews may not provide reliable results on the effects of MSPs on income and production. However, the findings were corroborated with data from validation workshops and post-project interviews by external evaluators, during which respondents shared similar information. This strengthened the findings to the extent that we feel confident about the conclusions despite the lack of data on, for instance, increased yields or income.

Second, lengthy field visits or large samples were not feasible due to limited budget and capacity. Furthermore, COVID-19 disrupted the fieldwork in the project’s final year, as travel to and from Ghana was no longer possible. However, a local PhD researcher involved in the Inclusive VCC project (the second author of this paper) organised and facilitated the final workshops to disseminate and validate the research results. In retrospect, this positively impacted the validation procedure as meeting participants could speak in their local language (Twi) without the interference of an interpreter and without feeling that they had to please the donor (whom they associated with ‘the white people’). The facilitator’s impression was that this made it easier for the participants to share their views openly.

Finally, it should be noted that based on the research conducted, it is not possible to draw conclusions about the long-term effects of MSPs, such as their impacts on farmers’ livelihoods, institutional changes, and environmental changes. This is a common limitation of agricultural and food-related MSPs in sub-Saharan Africa, as Van Ewijk and Ros-Tonen (2021) reported.

### Reflection on the Role of Engaged Scholars

A common feature of many agriculture and food-related MSPs is that researchers play a vital role in bringing together the actors involved.^24^ A systematic review of agricultural and food-related MSPs in sub-Saharan Africa has revealed that researchers frequently serve as facilitators or knowledge brokers in such initiatives (Van Ewijk & Ros-Tonen, 2021; Van Paassen et al., 2014). However, researchers can also take on other roles. For example, they can conduct scoping studies before platform meetings (e.g. Adjei-Nsiah & Klerkx, 2016; Triomphe et al., 2013; Vissoh et al., 2017), influence themes, engage actors, and shape the agenda of MSPs (Cullen et al., 2014; Musvoto et al., 2015), and participate in monitoring and evaluation (Dabire et al., 2017; Mulema et al., 2017).

Researchers also played a crucial role in the farmer-centred MSPs by generating insights into farmers’ realities, identifying innovations ‘from below’, facilitating knowledge exchange, building trust, and mediating power imbalances. Other scholars have also described these multiple roles in MSPs (e.g. Kilelu et al., 2014; Klerkx et al., 2013; Makate & Mango, 2017; Vissoh et al., 2017), but the farmer-centred approach to research had unique features. The PhD researchers and practitioners were familiar with the study area and smallholder farmers, which helped them understand farmers’ lifeworld and choose platform themes that reflected their main challenges. This approach enabled farmers to easily relate to these themes. Moreover, the Ghanaian PhD researchers were well-versed in the local culture and language, which was crucial in building solid relationships and trust with the farmers. These factors are essential in knowledge co-creation processes in MSPs (Makate & Mango, 2017; Vissoh et al., 2017) and emphasise the importance of good facilitation and leadership (Kusters et al., 2018; Van Paassen et al., 2014).

The authors were all involved in ‘a cyclical process of fact-finding, action, reflection, leading to further inquiry and action for change’ (Minkler, 2000, p.191). In this process, the researchers not only observed the activities and participants but also engaged in activities themselves (Spradley, 1980). This approach implies that our own and colleagues’ practices were part of the research, making it difficult to draw clear dividing lines between data collected ‘as part of the research’ and data available to the researcher ‘as part of the job’ (Somekh, 1995, p. 342). This grey area potentially raises an ethical issue, but we followed all ethical research principles, including informed consent, and made our position as researchers explicit from the beginning. We acknowledge that our position as engaged scholars had both advantages and limitations during data collection.

## Conclusion

This paper analysed the experiences of a transdisciplinary research project in Ghana that employed a farmer-centred approach to mobilise farmers and actors working with them in MSPs for inclusive knowledge exchange and co-creation, facilitating learning. Based on our findings, we conclude that the farmer-centred MSPs studied represent a genuinely different approach from most MSPs described in the literature. We discussed five design principles that give the iteratively developed approach a more solid conceptual basis: multiple functions (design principle 1), farmer-centred research (design principle 2), farmer-centred theme setting (design principle 3), creating a safe learning space (design principle 4), and local ownership of platform principles (design principle 5). These farmer-centred principles can be adopted in other geographical settings as they are not context-specific. However, the principles need to consider a region’s specific context.

These principles ensured that the approach was inclusive in various ways. First, the knowledge exchange was effectively aligned with farmers’ varied livelihood orientations, challenges, knowledge, experiences, capabilities, and innovation capacity, which is part of the *social dimension of inclusive development*. This was achieved by organising events close to the farmers’ lifeworld, actively identifying and documenting their knowledge and innovations ‘from below’, and making these accessible. The farmer-centred research and theme setting played a crucial role in this regard.

Second, the farmer-centred approach contributed to farmers’ empowerment, which is part of the *relational dimension of inclusive development*. A safe and empowering learning space was created by ensuring that at least half of the participants were farmers and that their knowledge and innovation capacity were foregrounded. Farmers felt comfortable sharing their knowledge and innovations, expressing their concerns, engaging with researchers, and opposing private sector and institutional actors. This facilitated the adoption of low-tech and low-cost innovations they learned from their peers, including some that led to their mobilisation in saving and credit groups.

Third, many practices adopted, particularly tree planting, applying organic manure and mulching, were in line with sustainability and landscape concerns, which is part of the *environmental dimension of inclusive development*.

We attribute the receptivity of the approach among farmers and some institutional actors to several factors, including (i) farmers being the majority of MSP participants, (ii) facilitation by researchers who spoke the local language, (iii) organising the meetings at a level close and relevant to farmers (district), (iv) concrete communication of previously identified innovations ‘from below’ (the tangibility rule), and sharing farmers’ knowledge and innovations during the meetings, and (v) bringing institutional actors together who usually do not communicate and collaborate much.

Despite some local institutional actors adopting farmer-centred platform principles, the organisation of farmer-centred MSPs still relies on external donor funding, making it challenging to institutionalise the approach at the district level. Future research could investigate ways to establish stronger links with national government bodies to incorporate farmer-centred approaches into mainstream policies. Additionally, further research is needed to identify entry points for institutionalising the farmer-centred approach to MSPs that help reduce donor dependency and facilitate upscaling. Finally, it is important to explore whether the farmer-centred approach and principles can be applied in other contexts.

We recommend that government agencies at the national and district level incorporate farmer-centred design principles into their work plans and budgets. Farmer-centred MSPs can contribute to inclusive learning and knowledge co-creation if institutional actors prioritise farmers’ knowledge and innovation capacity.

## Supplementary Information

Below is the link to the electronic supplementary material.

Supplementary file1 (DOCX 38 KB)

## Data Availability

Most project documents used in this paper are available on the project websites (https://puttingheadstogether.wordpress.com/; https://inclusivevcc.wordpress.com/; https://treefarms.wordpress.com/). The anonymised interview transcripts can be made available upon substantiated request.

## Footnotes

1 Context-embedded or experiential knowledge comprises local craft- and practice-based knowledge and sector-specific technical knowledge (Chambers, 1997; van Ewijk & Baud, 2009; Somuah et al., 2021).

2 Institutional actors include representatives of government agencies, public institutions such as the Ghana Cocoa Board (COCOBOD), Oil Palm Research Institute (OPRI), and Resource Management Support Centre (RMSC) of the Ghana Forestry Commission), and NGOs such as Solidaridad, the Rural Development Youth Association (RUDEYA), and Agribusiness in Sustainable Natural African Plant Products (ASNAPP).

3 Inclusive value chain collaboration (VCC) project. See https://inclusivevcc.wordpress.com/ for details on organisations involved in the project.

4 For more information see: https://puttingheadstogether.wordpress.com/ and https://www.nwo.nl/en/researchprogrammes/science-using-research-sure.

5 For project information, see https://treefarms.wordpress.com/.

6 In addition, the project had a spatial component to examine the effects of tree-crop expansion on landscape dynamics and the provision of ecosystem and landscape services (Asubonteng, 2022).

7 The project was also carried out with macadamia and avocado farmers in South Africa, but the farmer-centred approach was mainly developed in Ghana.

8 Accessed on 18 July 2021.

9 It should be noted that the interview guide does not make explicit reference to the design principles discussed in this paper as these were the result of ex-post conceptualisation in an effort to put the experience in a theoretical perspective and develop an analytical framework to assess the inclusivity of knowledge exchange in MSPs.

10 Farmers distinguished between landless farmers (sharecroppers and caretakers), farm owners, entrepreneur farmers, part-time farmers, and absentee farmers. All these groups were included in the learning platforms except for the absentee farmers as they usually lived outside the farming community.

11 Chief farmers are influential people in Ghana. They act as a source of information, as a contact person for the implementation of government programmes such as the mass spraying programme of the Ghana Cocoa Board (COCOBOD). The title of chief farmer is assigned to an influential person in a farmer community. They represent their community at district meetings, where regional chief farmers are elected. The latter are part of the Ghana Cocoa, Coffee, and Shea nut Farmers Association (CCSFA), which the government recognises as the legitimate negotiation partner on behalf of all cocoa, coffee, and shea nut producers (Quarmine, 2015). Although mainly documented in the cocoa, coffee, and shea nut sectors, we observed that there was also an influential chief farmer representing oil palm farmers in Kade.

12 Biases could emerge from (i) being actively engaged in the activities and data collection depending on opportunities (e.g. note-taking during meetings or informal talks) (Somekh 1995) and (ii) participant bias whereby institutional actors and farmers give answers they think will please the researcher.

13 Parts of the results draw from a draft paper presented by the authors at the Africa Knows! Conference organised by the African Studies Centre in Leiden in January 2021.

14 The recommended number in manuals and guides for cocoa extension in Ghana is 15–18 trees (see also Kaba et al., 2020).

15 The Ghana Forestry Commission pilots the registration of trees planted on farms because of the conflicts arising from the fact that farmers are only allowed to fell trees on their land that they planted themselves. Naturally regenerated trees, including those on farmland, fall under the custody of the Forestry Commission, which can issue permits to registered timber operators to harvest these trees (Ros-Tonen & Derkyi, 2018; Gaither et al., 2019).

16 Interviews with representatives of the Ministry of Food and Agriculture (MoFA), the Ghana Cocoa Board (COCOBOD), the Ghana Agricultural Associations Business and Information Centre (GAABIC) in October 2019 and the NGO Rural Development and Youth Association (RUDEYA) in June 2018.

17 Examples of such low-cost and accessible innovations ‘from below’ are time-saving techniques to husk the oil palm fruits, and bird-scaring techniques to prevent birds from eating the rice when it starts hassling. Social and institutional innovations were also reported, such as establishing saving groups, women associations, and informal partnerships to introduce a new crop. More examples can be found in a brochure available on https://inclusivevcc.files.wordpress.com/2018/07/learningplatform_brochure.pdf.

18 See data collection and sampling methods section for information on how the learning platform participants were selected.

19 Initially, the project aimed to follow and link with existing innovation platforms, such as those created by the Convergence of Sciences project (Röling et al., 2010; Nederlof & Pyburn, 2012). In the course of the project, thanks to an additional subsidy from the Lindt Cocoa Foundation, developing a different, more farmer-centred, approach became more prominent in the project.

20 See https://inclusivevcc.wordpress.com/publications/.

21 https://inclusivevcc.files.wordpress.com/2018/07/learningplatform_brochure.pdf.

22 The executive committee in Tepa included members from participating institutions such as the Cocoa Health and Extension Division (CHED) of the COCOBOD, the Ministry of Food and Agriculture, cocoa buyers like the Produce Buying Company (PBC Ltd) and Armajaro Ghana Limited (AGL, now AgroEcom), a Chief Farmer, and some purchasing clerks. In Kade, members included representatives of OPRI, a palm-oil company, MOFA, the Ghana Agricultural Associations Business and Information Centre (GAABIC), and an oil-palm Chief Farmer.

23 Other consortium parners were the Rural Development Youth Association (RUDEYA) and the University of Amsterdam, with the Ministry of Food and Agriculture and the NGO Agribusiness in Sustainable Natural African Plant Products (ASNAPP) being associated partners.

24 Aside from MSPs initiated and led by researchers. MSPs can also be local-led, in which case farmers and practitioners play a steering role (Warner, 2006; Van Ewijk & Ros-Tonen, 2021; Audouin et al., 2021).
