# Supplementary material for: Farmer-Centred Multi-stakeholder Platforms: From Iterative Approach to Conceptual Embedding
Source: J Knowl Econ. 2024 Feb 8;15(4):17077–107. doi: 10.1007/s13132-023-01661-7 (PMC11852531; doi:10.1007/s13132-023-01661-7)
Supplement: Supplementary file 1 — Supplementary file1 (DOCX 38 KB) [file 13132_2023_1661_MOESM1_ESM.docx]

**Supplementary material 1.**

**Interviewees and interview guide used for -depth interviews with project partners and lead actors**

1. **Interviewees**

***Inclusive Value Chain Collaboration Project***

*Accra and Kumasi*

- Agro Eco Louis Bolk Institute (NGO) 22-10-1019
- UENR – University of Energy and Natural Resources 21-10-2019

29-10-2019

- GAABIC – The Ghana Agricultural Associations Business and Information Centre

29-10-2019

- RMSC – Resource Management Support Centre of the Forestry Commission 25-10-2019

*Kade*

- OPRI – The Oil Palm Research Institute - 1 28-10-2019
- OPRI – The Oil Palm Research Institute - 2 28-10-2019
- Forestry Commission 28-10-2019
- GOPDC – Ghana Oil Palm Development Company (former employee) 30-10-2019

*Kumasi*

- COCOBOD – The Ghana Cocoa Board 29-10-2019
- MoFA – The Ministry of Food and Agriculture 26-10-2019

*Tepa*

- AGL – AGROECOM Ghana Limited (Licensed (cocoa) Buying Company 31-10-2019
- Chairman, learning platform committee 31-10-2019
- Chief farmer 31-10-2019
- MoFA – The Ministry of Food and Agriculture 1-11-2019
- COCOBOD – The Ghana Cocoa Board 1-11-2019

***Treefarms project***

- ASNAPP − Agribusiness in Sustainable Natural African Plant Products (NGO) 10-6-2018
- RUDEYA − Rural Development and Youth Association (NGO) 10-6-2018

12-6-2018

- RMSC − Resource Management Support Centre of the Forestry Commission 11-6-2018

12-6-2018

14-6-2018

- MoFA – The Ministry of Food and Agriculture 12-6-2018
- UENR – The University of Energy and Natural Resources 14-6-2018

1. **Interview Guide partners Inclusive Value Chain Collaboration Project Ghana October 2019**

**Introduction**

[personal introduction]

The Inclusive VCC project has come to an end. In this interview, we would like to obtain your ideas about this project, particularly your views on the collaboration between various partners and the process of knowledge exchange and knowledge co-creation, which was a central aspect of the project. By knowledge co-creation, we mean processes of knowledge sharing and joint learning between researchers, policymakers, practitioners and farmers, as well as researchers with various disciplinary backgrounds. Through this interview, we want to find out how the project partners perceive the collaboration between the various partners, the knowledge exchange processes, if and what they have learned from it, and if and how this idea of knowledge co-creation has affected the role and practices of the project partners.

[The interview will take approximately one hour. The data will be used as an input for the final report as well as for my postdoc research which builds on two NWO-WOTRO funded projects [already mentioned at the start of the interview]. The data will be used anonymously, although the names of the project partners will be included in the project documents. In case we would like to use a specific quote, we will first consult you. Do you mind if I record the interview? The recordings will be treated confidentially and will be erased after the transcriptions are finalised.

**I. General questions**

1. Name interviewee:

2. Organisation:

3. Affiliation/ position:

4. How do you see the role of your organisation in the project?

5. How do you see your own role in the project?

6. What was the motivation of your organisation to participate in the Inclusive VCC project?

7. What was your personal motivation to participate in the Inclusive VCC project?

8. Have there been changes within your organisations in the course of the project which have had an impacts on the roles and motivations?

**II. Experience with capacity building / knowledge sharing**

9. What is your organisation’s experience with knowledge sharing and capacity building processes? (probe: what kind of activities, target group, how often, how many people?)

10. What is your organisation’s experience in collaborating with different partners: in terms of having a different professional background and in terms of having a different disciplinary background?

**III. Capacity building and knowledge sharing in the Inclusive VCC project**

11. The Inclusive VCC is foremost a research programme aiming at knowledge exchange between researchers, practitioners and farmers. Have there been any activities in the Inclusive VCC project that you perceived as different than usual? If so, which ones? In what ways were they different?

12. Has the Inclusive VCC project in any way changed your way of thinking about knowledge sharing and capacity building? If yes: how? If no: why not?

**IV. Learning processes**

13. Did you learn something from the project? If so, what did you learn (probe: can you give examples of what you have learned from the project?) From who and when have you learned this? (probe: at what occasion?)

14. In case you learned something, did you apply lessons learnt? If so, what did you do with the newly acquired knowledge?

15. Did anything change in your work as a result of the project? (probe: can you give one or two examples?)

16. Was there any unforeseen learning? Something you had not expected to learn?

17. What do you think that others might have learned from you in this project? (probe: you might have had some feedback? Who has learned what from you?)

18. In what way do you usually prefer to obtain knowledge (e.g. workshops, meeting, reports, internet, ….)?

**V. Knowledge sharing in and outside the own organisation**

19. Did you share what you have learned with someone within your organisation?

20. Did the new knowledge result in new practices or ‘ways of doing things’ within your organisation? (Probe: Can you give an example? Has this been documented?)

21. Did you share what you have learned with someone from another organisation? How?

**VI. Potential and challenges**

22. The Inclusive VCC required collaboration between partners with a different professional background as well as project partners with a different disciplinary background originating from different countries. Do you experience advantages of such collaboration? Which ones?

23. What in your view are the challenges of such collaboration? How do you think that these challenges can be overcome?

24. Do you see any specific results coming out from the Inclusive VCC project? (Probe: If so, is this related to the knowledge co-creation process that WOTRO advocates?)

25. Do you expect there will be a lasting outcome of the Inclusive VCC project? Do you expect any project activity to continue after the project comes to a close?

**Interview Guide partners and farmers Treefarms June 2018**

**Introduction**

The Treefarms project was set up based on the idea of knowledge co-creation. By knowledge co-creation we mean processes of knowledge sharing and joint learning between researchers, policymakers, practitioners and farmers that lead to new knowledge and new ways of sharing knowledge. Through this interview we want to find out how the project partners and beneficiaries perceive the knowledge exchange processes, what they have learned from it, and if and how this idea of knowledge co-creation has affected the role and practices of the project partners

**I. General questions**

1. Name interviewee:

2. Organisation:

3. How do you see the role of your organisation in the project?

4. How do you see your own role in the project?

5. What was the motivation of your organisation to particpate in the Treefarms project?

**II. Experience with capacity building / knowledge sharing**

6. What is your organisation’s experience with capacity building and knowledge sharing processes? (probe: what kind of activities, target group, how often, how many people?)

7. How has your organisation generally dealt with capacity building and knowledge sharing?

(probe: standard procedure and setup? way of working? sharing what kind of knowledge?

8. Has the Treefarms project changed your way of thinking about capacity building and knowledge sharing? If yes: how? what do you differently now?

**III. Capacity building and knowledge sharing in the Treefarms project**

9. What do you see as capacity building and knowledge sharing activities in the Treefarms project?

10. In these activities, whose knowledge is exchanged, in your view? (who exchanges knowledge with whom?)

11. Have there been any activities that you perceived as different than usual? Which ones? Why were they different?

**IV. Learning processes**

12.What have YOU learned in the course of this project? (probe: can you give examples of what you have learned from the project? what kind of knowledge? from who have you learned that? how? when? at what occasion?)

13. What did you do with the newly acquired knowledge?

14. Did anything change in your work as a result of the newly acquired knowledge? (probe: can you give one or two examples?)

15. Was there any unforeseen learning, something you had not expected to learn?

16. What do you think that others have learned from you in this project? (probe: who has learned what from you? what has that knowledge been used for?)

17. In your view, have these learning processes resulted in new ideas, knowledge or ways of doing things? (probe: can you give one or two examples? what kind of knowledge/ideas? used for what? what kind of practices or ‘ways of doing things’?)

18. In what way do you usually prefer to obtain new knowledge (e.g. workshops, meeting, reading, internet, others)?

**V. Knowledge sharing**

19. Did you share what you have learned with someone? With whom? (probe: within the organisation, with another organisation, with organisations at national or international level?). How?

20. Did the new knowledge result in new practices or ‘ways of doing things’ within your organisation? Can you give an example? Has this been documented?

22. Are you aware of changes in practices or ‘ways of doing things’ in other organisations with whom this knowledge was shared?

**VI. Potential and challenges**

23. Normally projects do not so explicitly require collaboration between researchers, practitioners, policymakers and farmers. What in your view are the challenges of such collaboration?

24. How do you think that these challenges can be overcome?

25. Do you also experience advantages of such collaboration? Which ones?

26. Do you see any specific results coming out from the Treefarms project related to the knowledge co-creation process that WOTRO advocates?

27. What do you expect to be a lasting outcome of the Treefarms project? Do expect any project activity to continue after the project comes to a close?

**Supplementary material 2.**

**Research to identify innovations ‘from below’ in five steps**

**STEP 0 – Select a theme that is closely related to farmers’ lifeworld prior to the fieldwork**

**STEP 1 – Start the fieldwork by introducing the community**

On the first day, the researchers should introduce themselves to the village chief and opinion leaders to explain the research objectives and gain a preliminary understanding of village life, income-generating opportunities in the community, and recent changes and challenges related to the selected theme. Next, the leaders show the team around, using the transect walk technique, to enable the researchers to observe some of the community’s characteristics. The opinion leaders should also select the first group of focus group participants (community members), ensuring a mix of age and sex. These participants should be invited to participate on the next day.

**STEP 2 – Document changes**

The insights from the first day should be used to fine-tune the probing questions for the focus group discussion (FGD). Understanding community members’ perspectives on the theme and the meaning they attribute to it is crucial. Based on that understanding, the team can begin asking questions about the recent changes farmers observed concerning the theme. The FGD should also validate the findings and observations made on the first day. The rest of the day should be used for spontaneous interviews, observations, and processing of the day’s findings to present to new FGD participants the following day.

**STEP 3 – ‘Pains and Gains’^^[[1]](#footnote-1)^^**

On the third day, two focus groups should be held: one for men only and one for women only. This allows for capturing potential differences in perceived changes and impacts between male and female farmers, who have different responsibilities, needs, and interests and face different opportunities and constraints. It also creates a potentially safer space for women and men to speak up in the group. This third day should again begin with a validation of the findings of the previous day, allowing the groups to complement or bring in nuances. The focus should be on identifying the main problems (‘pains’) and opportunities (‘gains’) related to the discussed change. These ‘pains and gains’ are potential triggers for innovation, as community members may employ new ways using their own initiatives and resources to relieve their ‘pains’ and create their ‘gains’. On the same day, the researchers should facilitate ranking the main problems and challenges and try to get the first sense of existing strategies or ideas to reduce or overcome these challenges. The data obtained so far will be analysed to identify innovations and changemakers.

**STEP 4 – Identify innovations and changemakers**

The identification of innovations on day 3 should be followed up with interviews with community members or institutions that either use the new approach/technique or who were the actual ‘innovators’ (changemakers). If the previous days did not prompt any innovations, day four could be used for an additional mixed FGD to discuss how community members have responded to challenges and opportunities. The research team can inquire if they know someone who has developed new ways of dealing with challenges. This may require a lot of probing. If cases were identified, follow-up interviews and field visits are scheduled to find out the details of the innovation.

**STEP 5 – Document the innovation**

The last day consists of visits and in-depth interviews with the identified changemakers. The purpose is to get details of the innovation and document it with a photo or video camera to allow the tangible sharing of the innovation ‘from below’ during the learning platform meeting.

In the final step of the process, the research team aims to ensure a good gender balance in selecting changemakers who will attend the farmer-centred learning platform. The focus is on prioritising stories that have the potential to benefit other farmers. The selected innovations will be briefly presented at the platform meeting, which should be organised shortly after the field research by the research team. PowerPoint slides, pictures, and videos will be used to enhance the presentation. During the platform meeting, farmers who have implemented the innovations will be interviewed on stage.

1. This step draws from the Business Model Canvas (<https://www.strategyzer.com/canvas/business-model-canvas>). [↑](#footnote-ref-1)
